# Complete Genome Sequence of Undomesticated *Bacillus subtilis* Strain NCIB 3610

**DOI:** 10.1128/genomeA.00364-17

**Published:** 2017-05-18

**Authors:** Taylor M. Nye, Jeremy W. Schroeder, Daniel B. Kearns, Lyle A. Simmons

**Affiliations:** a Department of Molecular, Cellular and Developmental Biology, University of Michigan, Ann Arbor, Michigan, USA; b Indiana University, Department of Biology, Bloomington, Indiana, USA

## Abstract

*Bacillus subtilis* is a Gram-positive bacterium that serves as an important experimental system. *B. subtilis* NCIB 3610 is an undomesticated strain that exhibits phenotypes lost from the more common domesticated laboratory strains. Here, we announce the complete genome sequence of DK1042, a genetically competent derivative of NCIB 3610.

## GENOME ANNOUNCEMENT

Bacterial strains passaged in the laboratory have lost several phenotypes that are readily observable with “wild” or undomesticated strains (1–3). *Bacillus subtilis* strain NCIB 3610 (abbreviated as 3610) is a derivative of Marburg with genomic similarity to *B. subtilis* 168 (4). Undomesticated 3610 forms multicellular structures with complex architecture and cell organization (1, 2). Pellicle biofilms, swarming motility, exopolysaccharide capsule, surfactant, and the production of antimicrobials are just a few examples of the many phenotypes that have been lost from laboratory strains (4–9). In addition to the differences in phenotypes, 3610 also harbors an 84 kb plasmid (pBS32) that has been cured from domesticated strains. Plasmid pBS32 encodes 102 genes including *comI*, the product of which is an inhibitor that prevents 3610 from developing genetic competence (10). The recent identification of ComI as a genetic competence inhibitor allowed for the generation of the naturally competent 3610 *comI*^Q12L^ (DK1042) strain, facilitating genetic study of the complex phenotypes associated with undomesticated strains (10). To further expedite genetic studies, we report the completed reference genome for DK1042, where the *comI*^Q12L^ point mutation is the only known mutation in an otherwise 3610 genetic background. Our results provide a publicly available complete reference genome for a competent 3610 derived strain, helping to forward the use of 3610 as a genetic platform for studying phenotypes and behaviors not present in domesticated laboratory strains.

Genomic DNA from DK1042 (NCIB 3610 *comI*^Q12L^) (10) was purified via phenol-chloroform extraction (11). Sequencing libraries were prepared with 5-kb mean insert size for sequencing on the Pacific Biosciences (PacBio) RS II sequencer by the University of Michigan Sequencing Core. Two single-molecule real-time cells were used to sequence the libraries. The average subread length was 3.5 kb. *De novo* genome assembly was performed using RS_HGAP_Assembly.3 version 2.3.0 (12), resulting in two contigs representing the chromosome and plasmid (pBS32). A break was introduced at the chromosomal origin *in silico* and the genome was circularized using the minimus2 script from AMOS (13). The original PacBio sequencing reads were remapped to the circularized reference genome via RS_Resequencing.1, resulting in a consensus accuracy of 99.9997% and chromosomal coverage of 625. The previously sequenced pBS32 plasmid sequence [accession no. KF365913 (10)] was then added as a second contig to create the final NCIB 3610 *comI*^Q12L^ reference genome. The final reference genome consists of a 4,215,607 bp chromosome and 84,215 bp plasmid. The 2 contigs have a combined length of 4,299,831 bp. The previous reference genome for NCIB 3610 totaled 4,292,969 bp (chromosome and plasmid) and consisted of 84 contigs (14). Thus, the reference genome presented here has reduced the number of contigs from 84 to 2, with a 6.8 kb longer sequenced genome size.

### Accession number(s).

Gene annotation was performed using the Prokaryotic Genomes Annotation Pipeline (PGAP) through NCBI. The complete genome sequence of *B. subtilis* subsp. *subtilis* strain NCIB 3610 *comI*^Q12L^ was deposited in the DDBJ/EMBL/GenBank database with accession no. CP020102 for the chromosome and CP020103 for pBS32 (10).
